# RAD51AP1在肿瘤进展和耐药中的研究进展

**DOI:** 10.3779/j.issn.1009-3419.2023.102.34

**Published:** 2023-09-20

**Authors:** Renwang LIU, Mingbiao LI, Zixuan HU, Zuoqing SONG, Jun CHEN

**Affiliations:** ^1^300052 天津，天津医科大学总医院肺部肿瘤外科; ^1^Department of Lung Cancer Surgery, Tianjin Medical University General Hospital; ^2^天津市肺癌转移与肿瘤微环境重点实验室，天津市肺癌研究所; ^2^Tianjin Key Laboratory of Lung Cancer Metastasis and Tumor Microenvironment, Tianjin Lung Cancer Institute, Tianjin 300052, China

**Keywords:** RAD51AP1, 肿瘤干细胞, 耐药, RAD51AP1, Cancer stem cells, Drug resistance

## Abstract

基因组失稳可能导致多种肿瘤的发生发展。同源性重组修复（homologous recombination repair, HRR）是机体维持基因组稳态的重要机制之一。RAD51相关蛋白1（RAD51 associated protein 1, RAD51AP1）在HRR中至关重要，其主要参与D-loop的形成，是维持细胞基因组稳态的重要分子。然而，据文献报道RAD51AP1在多种癌种中显著高表达，并与预后呈负相关，提示其可能具有显著的促癌作用。其促癌作用机制尚不明确，可能与肿瘤干性密切相关。同时，RAD51AP1还在放疗和化疗的耐药中具有重要作用。探索RAD51AP1和其上下游调控机制可能能够找寻逆转肿瘤治疗耐药的新靶点。因此，我们综述了目前关于RAD51AP1在肿瘤中的研究，归纳了其在各种肿瘤中的差异表达及与预后的关系，总结了其促癌作用的潜在机制，分析了其促进耐药相关研究的现状，旨在为后续寻找抑制肿瘤进展和逆转耐药的靶点的研究提供一定的方向。

## Abstract (en)

The genomic instability may lead to an initiation of cancer in many organisms. Homologous recombination repair (HRR) is vital in maintaining cellular genomic stability. RAD51 associated protein 1 (RAD51AP1), which plays a crucial role in HRR and primarily participates in forming D-loop, was reported as an essential protein for maintaining cellular genomic stability. However, recent studies showed that RAD51AP1 was significantly overexpressed in various cancer types and correlated with poor prognosis. These results suggested that RAD51AP1 may play a significant pro-cancer effect in multiple cancers. The underlying mechanism is still unclear. Cancer stemness-maintaining effects of RAD51AP1 might be considered as the most reliable mechanism. Meanwhile, RAD51AP1 also promoted resistance to radiation therapy and chemotherapy in many cancers. Thus, researches focused on RAD51AP1, and its regulatory molecules may provide new targets for overcoming cancer progression and treatment resistance. Here, we reviewed the latest research on RAD51AP1 in cancers and summarized its differential expression and prognostic implications. In this review, we also outlined the potential mechanisms of its pro-cancer and drug resistance-promoting effects to provide several potential directions for further research.

肿瘤目前仍是全球范围内最常见的死亡原因之一，基因组失稳是其发生发展的重要机制之一^[1,2]^。外源性DNA损伤（如重金属、电离辐射等）和内源性DNA损伤（如氧自由基等）都可以导致细胞基因组失稳，从而使正常细胞获得恶性表型^[3]^。同源性重组修复（homologous recombination repair, HRR）等DNA损伤修复机制是机体维持基因组稳态的重要机制^[4]^。HRR的缺失可能导致卵巢癌、乳腺癌、胰腺癌等多种肿瘤的发生发展^[5]^。

HRR的生物学过程比较复杂^[6]^。简单来说，当细胞发生DNA损伤时，重组酶RAD51结合断裂的单链DNA（single-stranded DNA, ssDNA）后，形成突触前纤维，进而捕获供体DNA形成D-loop，以完成HRR^[6]^。RAD51相关蛋白1（RAD51 associated protein 1, RAD51AP1）作为重组酶RAD51的辅助蛋白，可以招募RAD51，并促进RAD51和单端侵入中间体（single-end invasion intermediate, SEI）DNA的结合，进而刺激D-loop的形成^[7]^。因此，RAD51AP1在HRR中具有重要作用，其可能通过促进HRR以维持细胞基因组稳态，进而减少肿瘤的发生。

然而，多项研究^[8⇓⇓-11]^表明，RAD51AP1在许多恶性肿瘤组织及转移灶中显著高表达，且其高表达往往提示不良预后。这些结果提示RAD51AP1可能具有促癌作用。其促癌机制尚不明确。同时，还有文献^[12,13]^报道，RAD51AP1亦有促进放化疗耐药的作用。RAD51AP1在肿瘤发生发展及耐药中的广泛作用提示其可能成为一个抑制肿瘤、逆转耐药的新靶点。

因此，本文综述了RAD51AP1在肿瘤中的研究，并聚焦于其促癌作用的潜在机制。同时，回顾总结了其在放化疗耐药中的作用，并对其未来在逆转靶向治疗耐药和肿瘤免疫治疗中的研究进行展望，旨在为后续研究提供一定的理论依据。

## 1 RAD51AP1的生物学特性

RAD51AP1于1997年由Mizuta等首次发现^[14]^。人RAD51AP1基因定位于染色体12p13区带^[15]^，其在脊椎动物中高度保守^[16]^。人RAD51AP1和小鼠Rad51ap1均主要分布在胸腺、骨髓、脾脏和睾丸中^[14,17]^，其细胞亚定位主要位于细胞核中^[18]^。

## 2 RAD51AP1在肿瘤中的促癌作用

RAD51AP1在多种肿瘤中显著高表达。早在2004年，Song等^[19]^应用互补DNA（complementary DNA, cDNA）微阵列芯片的方法首次报道了RAD51AP1在乙肝病毒阳性（hepatitis B virus positive, HBV+）的肝细胞癌中高表达，并应用反转录聚合酶链式反应（reverse transcription-polymerase chain reaction, RT-PCR）在肝细胞癌细胞株中进行了验证。此后，Henson等^[20]^和Matinez等^[21]^分别应用类似的方法发现RAD51AP1在人乳头状瘤病毒阳性头颈部鳞状细胞癌和套细胞淋巴瘤中均显著高表达。2007年，Martin等^[22]^首次应用一种类似于生信分析的方法，分析了van’t Veer等报道的结果，发现RAD51AP1在乳腺癌易感基因-1（breast cancer susceptibility gene 1, BRCA-1）缺失的乳腺癌中显著高表达。之后，各项研究发现，在肝内胆管细胞癌、卵巢癌、卵巢浆液性囊腺癌、口咽鳞状细胞癌、食管癌、结直肠癌、食管鳞状细胞癌、丙肝病毒阳性肝细胞癌及HBV+肝细胞癌中均有RAD51AP1的显著高表达^[8,9,13,17,23⇓⇓⇓⇓⇓⇓⇓-31]^。此外，切尔诺贝利事件后甲状腺乳头状癌（papillary thyroid cancer, PTC）即低剂量辐射暴露的PTC的RAD51AP1表达水平低于其他PTC水平，提示低剂量辐射的PTC可能存在不一样的致癌因素，其具体机制尚无报道^[32]^。

RAD51AP1的高表达往往提示患者预后不良。Sankaranarayanan等^[24]^应用Tensor GSVD方法，在生信层面对来源于肿瘤基因组图谱（The Cancer Genome Atlas, TCGA）数据库的249例卵巢浆液性囊腺癌患者资料进行分析，发现RAD51AP1高表达患者总生存期（overall survival, OS）显著低于低表达患者。其后，Chudasama等^[26]^通过收集患者组织和外周血样本，分析发现RAD51AP1高表达的卵巢癌和肺癌患者均表现出较低的OS。Zhao等^[33]^通过生信分析发现，在卵巢癌中，RAD51AP1高表达患者的OS及无进展生存期（progression-free survival, PFS）均较差。Zhuang等^[34]^应用同样的方法发现，肝细胞癌（hepatocellular carcinoma, HCC）中RAD51AP1的表达与OS及PFS亦显著负相关。Bridges等^[12]^通过生信分析发现，RAD51AP1高表达的乳腺癌患者不仅OS更差，其各种治疗后的无复发生存时间（recurrence free survival, RFS）也更低。此后，各项研究^[8⇓-10]^表明，在包括乳腺癌、肺腺癌等多种癌种中，RAD51AP1的高表达往往提示预后不良。因此，RAD51AP1可能是指导肿瘤的预后重要生物标记物。然而亦有研究^[29]^报道，在口咽鳞状细胞癌中尚未发现其与OS的相关性，而在Li等^[30]^的研究中也发现RAD51AP1高、低表达患者RFS的差异无统计学意义，在这些癌种中，RAD51AP1指导预后的作用仍需进一步的验证。

RAD51AP1介导肿瘤细胞的增殖、侵袭、迁移、抑制凋亡等多项表型的重要作用也在体内和体外实验中得到了验证。Obama等^[17]^最早在肝内胆管细胞癌的细胞株TFK-1和SSP25中敲除RAD51AP1后发现，其克隆形成能力明显下降，提示RAD51AP1对肝内胆管细胞癌细胞的增殖至关重要。Chudasama等^[26]^应用si-RAD51AP1转染SKOV3和A549细胞后发现细胞的增殖能力明显下降。Wu等^[27]^以非小细胞肺癌细胞株A549、H520为研究对象，敲减RAD51AP1后发现，肿瘤细胞的增殖、侵袭、迁移能力明显下降，凋亡细胞明显增多，并在裸鼠中做了进一步验证。

因此，RAD51AP1在多种肿瘤中显著高表达，其高表达提示患者预后不良。其还可以在多种肿瘤中促进肿瘤细胞的增殖、侵袭并抑制凋亡，具有广泛的促癌作用（表1）。

**表1:** RAD51AP1在肿瘤中的各项研究的材料、方法及结果

Authors	Cancer type	Clinical validation							Bioinformatics analysis					
		Specimens	Control	Methods	Expression	OS	PFS		Database	Size	Methods	Expression	OS	PFS
Pathania, et al.^[8]^	BC	N/A							TCGA GOBO	N/A	GOBO gene enrichment application, GSA tumor analysis	Overexpressed	Worse	N/A
Li, et al.^[9]^	LUAD	Combined with bioinformatics analysis							GEO, TCGA	2 datasets and 76 LUAD paired samples from the authors' department	RNAseq, Fragments Per Kilobase Million, RSEM, DESeq, Benjamini-Hochberg test	Overexpressed	Worse	N/A
Le, et al.^[10]^	TNBC	N/A							GEO, TCGA	165 tumors and 33 normals	"WGCNA", "EdgeR" and "Limma" packages in R	Overexpressed	Worse	N/A
Bridges, et al.^[12]^	BC	11 BCs	Matched normal tissues	PCR	Overexpressed	N/A	N/A		TCGA, UCGC, DAVID	456 tumors and 114 normals	N/A	Overexpressed	Worse	Worse
Bridges, et al.^[13]^	CRC	18 CRCs	Matched normal tissues	PCR	Overexpressed	N/A	N/A		TCGA, UCGC, DAVID, cBioportal	288 tumors and 41 normals	N/A	Overexpressed	Worse	N/A
Obama, et al.^[17]^	ICC	21 ICCs	10 normal intrahepatic bile duct epithelia samples	PCR, IHC	Overexpressed	N/A	N/A		N/A					
Song, et al.^[19]^	HCC	10 HCCs	Matched normal tissues	PCR	Overexpressed	N/A	N/A		N/A					
Henson, et al.^[20]^	MCL	5 MCLs	Other tumors and negative control	“virtual” northern	4 of 5 overexpressed	N/A	N/A		N/A					
Martinez, et al.^[21]^	HPV+ SCCHN	3 HPV+ SCCHN	4 normals	cDNA microarray	Overexpressed	N/A	N/A		N/A					
Martin, et al.^[22]^	BRCA1 (-) BC	N/A							Other researchers' publication	117 BCs	BRB array tools	Overexpressed in BRCA1 deficient BC than sporadic BC	N/A	N/A
Miles, et al.^[23]^	OC	N/A							TCGA	258 tumors and 6 normals	Principal component analysis, Consensus cluster	Overexpressed	N/A	N/A
Sankaranarayanan, et al.^[24]^	OV	N/A							TCGA	249 patients	Tensor GSVD	Overexpressed	Worse	N/A
Authors	Cancer type	Clinical validation							Bioinformatics analysis					
		Specimens	Control	Methods	Expression	OS	PFS		Database	Size	Methods	Expression	OS	PFS
Nguyen, et al.^[25]^	HCV+HCC	2 HCV+ HCCs	Matched normal tissues	Western Blotting	Overexpressed	N/A	N/A		N/A					
Chudasama, et al.^[26]^	OC, LC	5 OCs and 5 LCs	Matched normal tissues	PCR	Overexpressed	Worse	N/A		GEO	N/A	Glasso and Bayesian network (BN)	Overexpressed	N/A	N/A
Wu, et al.^[27]^	NSCLC	91 NSCLCs	Matched normal tissues	IHC	Overexpressed	N/A	N/A		GEO	4 datasets	"Limma" package in R	Overexpressed	N/A	N/A
Xie, et al.^[28]^	HBV+HCC	N/A							GEO	145 tumors and 154 normals	RobustRankAggreg package in R	Unknown but alteration	Worse	N/A
Chen, et al.^[29]^	LSCC	N/A							GEO, TCGA	2 Datasets from GEO and 111 LSCCs plus 12 matched non-cancerous samples from TCGA	"Limma" and "EdgeR" packages in R, Gene Ontology Enrichment Analysis	Overexpressed	NS	N/A
Li, et al.^[30]^	EC	N/A							GEO, TCGA	3 datasets	GEO2R and Venn diagram web tool, "RTCGAToolbox" package in R	Overexpressed	Worse	NS
Zheng, et al.^[31]^	ESCC	6 ESCCs	Matched normal tissues	PCR	Overexpressed	N/A	N/A		GEO, TCGA, UALCAN, sequence read archive for scRNA-seq data	6 datasets	“Limma” package in R	Overexpressed	Worse	N/A
Handkiewicz-Junak, et al.^[32]^	Post-chernobyl PTC	33 ECR PTCs	32 non-ECR PTCs	cDNA microarray, PCR	Lower expression in ECR tumors than non-ECR tumors	N/A	N/A		N/A					
Zhao, et al.^[33]^	OC	N/A							GEO, TCGA， UCSC Xena, Oncomine	3 datasets	"affy" package in R	Overexpressed	Worse	Worse
Zhuang, et al.^[34]^	HCC	N/A							GEO, TCGA	8 datasets	N/A	Overexpressed	Worse	Worse

OS: overall survival; PFS: progression-free survival; BC: breast cancer; SMM: syngeneic mouse models; N/A: not available; OC: ovarian cancer; CRC: colorectal cancer; ESCC: esophageal squamous cell carcinoma; LC: lung cancer; NSCLC: non-small cell lung cancer; HBV: hepatitis B virus; HCV: hepatitis C virus; HCC: hepatocellular carcinoma; TNBC: triple-negative breast cancer; NS: no significance; LSCC: laryngeal squamous cell cancer; EC: esophageal cancer; ICC: intrahepatic cholangiocarcinoma; MCL: mantle cell lymphoma; HPV: human papilloma virus; SCCHN: squamous cell carcinoma of the head and neck; OV: ovarian serous cystadenocarcinoma; PTC: papillary thyroid cancer; ECR: exposed to chernobyl radiation; PCR: polymerase chain reaction; IHC: immunohistochemistry; GEO: Gene Expression Omnibus; TCGA: The Cancer Genome Atlas; DAVID: The Database for Annotation, Visualization and Integrated Discovery; LUAD: lung adenocarcinoma.

## 3 RAD51AP1促癌作用的潜在机制

### 3.1 RAD51AP1通过提高肿瘤细胞干性促进增殖

RAD51AP1的促癌机制尚不明确。其可能通过提高肿瘤干性来促进肿瘤的增殖。如Pathania等^[8]^发现，联合应用DNA甲基转移酶（DNA methyltransferase, DNMT）和组蛋白去乙酰酶（histone deacetylase, HDAC）抑制剂可以显著降低乳腺癌干细胞数量，并通过测序发现，乳腺癌干细胞的RAD51AP1的转录水平明显降低，提示联用药物抑制肿瘤的干性的作用机制可能是经由抑制RAD51AP1介导的。随后，他们进一步研究^[12]^发现，RAD51AP1敲减后的乳腺癌细胞相关干性标记物CD44、CD49f、NANOG和KLF4等表达明显降低，肿瘤干细胞（cancer stem cells, CSCs）数目明显降低，CSCs的成球能力也明显降低，并在转基因小鼠和裸鼠中均进行了验证，证实了RAD51AP1通过提高肿瘤细胞的干性来促进增殖和转移。进一步，他们应用同样的方法验证了RAD51AP1在结直肠癌中也是通过类似的机制促癌发生发展^[13]^。另外，Redmer等^[35]^也发现，在CD271稳转的黑色素瘤细胞T20/02和A375中，RAD51AP1明显高表达，提示RAD51AP1可能与黑色素瘤干性维持作用密切相关。同时，我们的前期工作中，通过对泛癌种进行生信分析发现，RAD51AP1的表达在多个癌种中与干性指标mRNAsi评分和mDNAsi评分呈显著正相关，提示其可能具有广泛的提高肿瘤干性的作用。然而，RAD51AP1提高干性的作用并未在乳腺癌和结直肠癌以外的肿瘤中得到实验数据的证实。其维持肿瘤干性的机制也尚不明确，未来基于此研究可能有助于找寻抑制CSCs的新靶点。

### 3.2 RAD51AP1促癌发生发展的其他机制

RAD51AP1还可能通过其他机制促进肿瘤发生发展。例如，其可以通过刺激端粒延长代替机制（alternative lengthening of telomeres, ALT）以维持肿瘤细胞的端粒长度，从而促进肿瘤细胞的增殖。肿瘤细胞主要通过两种端粒维持机制（telomere maintenance mechanisms, TMM）来抑制端粒缩短。其中之一就是HRR介导的ALT，占所有肿瘤的10%-15%^[36,37]^。Barroso-Gonzalez等^[38]^研究发现，E3连接酶MMS21介导的RAD51AP1苏木化可以有效地维持其在ALT阳性肿瘤细胞中的稳定性，进而通过招募POLD3和RAD52蛋白后介导的断裂诱导端粒DNA合成（break induced telomere DNA synthesis, BITS）以维持ALT系统，延长端粒长度，促进增殖。同时，研究^[39]^表明，RAD51AP1还能够以RAD52非依赖形式上调ALT。其可以与端粒重复序列RNA（telomere repeat-containing RNA, TERRA）结合，在端粒形成R-loop，刺激G-四聚体（G-quadruplexes, G4s）的合成，进而上调ALT。Kaminski等^[40]^也发现，基于其自身的SUMO-SIM调节轴作用，RAD51AP1可以结合并稳定含有TERRA的R-loop，刺激端粒延长，进而促进肿瘤细胞增殖。

另外，还有研究^[33]^发现，在卵巢癌细胞中，siRAD51AP1后，SMAD2-4、TGFBR1转录水平明显下降，p-SMAD2、p-SMAD3表达明显下降，提示RAD51AP1可能通过转化生长因子-β（transforming growth factor-β, TGF-β）/Smad信号通路促进肿瘤增殖。同时，Chudasama等^[26]^通过敲减卵巢癌和肺癌细胞中的RAD51AP1后，发现干性相关蛋白SOX2、促转移相关蛋白SNAI1及哺乳动物雷帕霉素靶蛋白（mammalian target of rapamycin, mTOR）信号通路相关蛋白有不同程度的低表达，提示其可能通过mTOR信号通路介导增殖。然而上述两种机制的研究者仅分析了RAD51AP1与TGF-β/Smad和mTOR信号通路关键蛋白的表达相关性，并未对其分子相互作用等直接机制进行进一步的研究。未来基于RAD51AP1通过何种机制调控这两个下游信号通路的研究可能能够发现阻断其促进肿瘤增殖的靶点和方法。

### 3.3 上调RAD51AP1表达的机制

肿瘤中调控RAD51AP1表达的机制尚不明确。Wang等^[41]^研究发现，转录因子ZEB1是RAD51AP1的重要上游调控分子之一。该研究证实，miR-320a可以通过与RAD51AP1的3’UTR区域结合而抑制其翻译，而ZEB1可以结合miR-320a启动子中的E-box区域来抑制miR-320a的表达，从而促进RAD51AP1的表达。同时，RAD51AP1的苏木化/泛素化平衡对其表达也具有重要作用。Barroso-Gonzalez等^[38]^发现，在ALT阳性肿瘤细胞中，E3连接酶MMS21可以介导RAD51AP1的苏木化，以减少泛素化对其的降解作用，从而维持RAD51AP1蛋白的稳定表达。还有一些学者应用生信分析发现可能存在一些功能基因和microRNA（如has-miR-140-3p等）与RAD51AP1的表达密切相关，但这些研究均未对其进行进一步的体内外实验验证^[10,23,24]^。

### 3.4 小结

我们对RAD51AP1下游及上游相关调控机制的研究进行了综述。研究表明RAD51AP1主要通过提高干性、促进ALT及上调TGF-β/Smad和mTOR通路等机制促进肿瘤发生发展。在肿瘤中，其高表达主要经由ZEB1和MMS21调控。未来基于其调控机制的研究将有助于进一步的转化医学研究。

## 4 RAD51AP1与耐药

RAD51AP1的高表达还可以促进肿瘤对化疗和放疗的耐药。Obama等^[17]^应用γ-射线对肝内胆管细胞癌细胞进行照射后，检测到RAD51AP1在细胞核内显著聚集。Henson等^[20]^在Hela细胞中敲减RAD51AP1后，可以明显提高其对丝裂霉素（mitomycin C, MMC）的敏感性。Zhou等^[42]^发现，在多形性胶质母细胞瘤中，敲减RAD51AP1可以显著增强替莫唑胺的敏感性。Li等^[43]^证实，甲基转移酶3（methyltransferase-like 3, METTL3）通过上调RAD51AP1促进结直肠癌细胞的5-氟尿嘧啶（5-fluorouracil, 5-FU）耐药。

RAD51AP1促进耐药的机制尚不明确。Bridges等^[13]^发现RAD51AP1在5-FU耐药的结直肠癌细胞株中的表达明显高于敏感株，且耐药细胞株的克隆成球能力更强，干性分子标记物NANOG、KLF4、OCT4和SOX2表达水平更高，提示耐药可能与RAD51AP1介导的干性维持作用有关。同时，他们还发现在乳腺癌细胞中敲减RAD51AP1可以显著提高培美曲塞（paclitaxel, PTX） 和电离辐射介导的凋亡作用，并在耐药株CAL51-PTX-R中发现RAD51AP1显著高表达，敲减RAD51AP1可能通过降低其干性能力来逆转耐药^[12]^。CSCs在肿瘤放化疗耐药中具有重要作用^[44⇓-46]^。在肿瘤中，优势CSCs分化主导肿瘤的进展，而其他CSCs处于静默状态。化疗治疗后，优势肿瘤细胞被杀灭，而静默的CSCs通过筛选后得到发生发展的机会，进而引起耐药^[13]^。因此，RAD51AP1提高肿瘤干性的作用可能是其促进肿瘤放化疗耐药的重要机制之一。

基于RAD51AP1与耐药的研究可能具有较大的应用前景。Ni等^[47]^发现，放射处理的宫颈癌细胞中RAD51AP1明显高表达，且与剂量呈正相关。而转录调节因子BRD4的抑制剂JQ1可以抑制RAD51AP1的表达，进而增加放疗的敏感性。其主要机制是，BRD4直接结合RAD51AP1上游350 bp的启动子区域而促进RAD51AP1的表达，而JQ1通过抑制BRD4的表达，减少了RAD51AP1的转录。另外，高剂量硒化物可以与细胞毒化疗药物产生协同作用以达到一定程度的逆转耐药^[48]^。Song等^[49]^研究发现，在卵巢癌细胞中，卡铂可以显著提高RAD51AP1的表达，而联合应用亚硒酸时，RAD51AP1却明显降低，提示硒化物可能存在通过降低RAD51AP1的表达而中和细胞毒药物耐药的机制。

总之，RAD51AP1可以介导多种肿瘤的放化疗耐药，其耐药机制主要与其提高肿瘤干性密切相关。基于RAD51AP1介导放化疗耐药的研究可能具有较大的临床应用前景。

## 5 总结与展望

综上所述，RAD51AP1不仅在正常细胞的HRR中具有重要作用，还可能具有广泛的促癌作用。其在多种肿瘤组织中高表达，并提示不良预后。RAD51AP1主要通过提高肿瘤干性、延长端粒、调控TGF-β/Smad和mTOR信号通路等机制来促进肿瘤的增殖、侵袭及迁移。其提高肿瘤干性的能力也是其促进多种肿瘤放化疗耐药的主要机制之一。以RAD51AP1为靶点，对其上游和下游调控机制进行干预可能能够成为研究逆转肿瘤放化疗耐药的重要方向。

此外，RAD51AP1在靶向治疗耐药和肿瘤免疫治疗中的作用报道罕见。靶向治疗可以通过调节表观修饰、改变肿瘤微环境、上调药物转运蛋白等机制介导耐药^[50,51]^。联合CSCs抑制剂可以部分逆转肿瘤靶向治疗的耐药^[52]^。同时，CSCs还可以通过减少抗原呈递和上调Wnt、Notch等信号通路的方式促进肿瘤免疫逃逸^[53,54]^。靶向CSCs逆转免疫治疗耐药的治疗效果已在动物实验、预临床实验中得到了部分验证^[55,56]^。而RAD51AP1可以显著提高肿瘤中CSCs的数量和干性能力，未来基于RAD51AP1的研究可能能够为逆转靶向治疗耐药和增效免疫治疗疗效的研究提供重要的参考。

Competing interests

The authors declare that they have no competing interests.
